# The efficacy of centralized nursing management based on risk prevention and control on the outcomes in children undergoing bronchoscopic interventional therapy

**DOI:** 10.12669/pjms.42.6.14692

**Published:** 2026-06

**Authors:** Yuhui Cui, Wenya Wei, Peiqing Huo, Lina Huang, Yanhua Wang

**Affiliations:** 1 Yuhui Cui, Department of Respiratory 2, Children’s Hospital of Hebei Province, Shijiazhuang, Hebei Province 050031, P.R. China. Hebei Provincial Clinical Research Center for Child Health and Disease, Hebei Provincial Medical Key Discipline, Shijiazhuang, Hebei Province 050031, P.R. China; 2 Wenya Wei, Department of Respiratory 2, Children’s Hospital of Hebei Province, Shijiazhuang, Hebei Province 050031, P.R. China. Hebei Provincial Clinical Research Center for Child Health and Disease, Hebei Provincial Medical Key Discipline, Shijiazhuang, Hebei Province 050031, P.R. China; 3 Peiqing Huo, Department of Respiratory 2, Children’s Hospital of Hebei Province, Shijiazhuang, Hebei Province 050031, P.R. China. Hebei Provincial Clinical Research Center for Child Health and Disease, Hebei Provincial Medical Key Discipline, Shijiazhuang, Hebei Province 050031, P.R. China; 4 Lina Huang, Department of Respiratory 2, Children’s Hospital of Hebei Province, Shijiazhuang, Hebei Province 050031, P.R. China. Hebei Provincial Clinical Research Center for Child Health and Disease, Hebei Provincial Medical Key Discipline, Shijiazhuang, Hebei Province 050031, P.R. China; 5 Yanhua Wang, Department of Nursing, Children’s Hospital of Hebei Province, Shijiazhuang, Hebei Province 050031, P.R. China. Hebei Provincial Clinical Research Center for Child Health and Disease, Hebei Provincial Medical Key Discipline, Shijiazhuang, Hebei Province 050031, P.R. China

**Keywords:** Bronchoscopic Intervention, Centralized Nursing Management, Risk Prevention and Control

## Abstract

**Objective:**

To explore the value of centralized nursing management based on risk prevention and control in bronchoscopy intervention therapy.

**Methodology:**

In this single-center retrospective comparative study, the clinical data from 200 pediatric patients undergoing bronchoscopy intervention in Children’s Hospital of Hebei Province from January 2023 to December 2024 was conducted. The patients were divided into two groups according to the nursing plan. The control group received routine nursing care, while the study group received centralized nursing management based on risk prevention and control. Both groups received one week of intervention. Treatment compliance was compared using Chi-square, while vital signs were compared using t-test.

**Results:**

The treatment compliance of the study group was higher than that of the control group (P<0.05). After intervention, the vital signs of the study group were better than those of the control group (P<0.05). The number of bronchoscopic procedures, hospitalization costs, and length of hospital stay were lower in the study group than in the control group (P<0.05). The incidence of complications was numerically lower and family satisfaction was numerically higher in the study group than in the control group, although these findings should be interpreted cautiously because of the small number of events.

**Conclusion:**

Centralized nursing management based on risk prevention and control was associated with improvements in selected short-term outcomes, including treatment compliance, vital-sign stability, and healthcare resource utilization, in children undergoing bronchoscopic interventional therapy.

## INTRODUCTION

Bronchoscopic interventional therapy is a key diagnostic and therapeutic method for intractable respiratory diseases in children.^1^ Although bronchoscopy is a minimally invasive procedure, clinical nursing of children is still challenging due to immature physiological development, narrow airway diameter, poor cooperation, and susceptibility to perioperative complications like airway spasm, bleeding, infection, and agitation.^2^–^4^ Currently, nursing care for pediatric bronchoscopic interventional therapy is primarily decentralized, with preoperative assessment, intraoperative cooperation, and postoperative monitoring conducted by different nursing teams. As a result, the efficiency of nursing is often affected by poor correlation of nursing procedures, delayed risk early warning, lack of systematic dynamic monitoring of children’s individual symptoms, and difficulty in achieving closed-loop management of “risk prediction - real-time intervention - disease outcome”.^5^–^7^

Gradual transformation of pediatric nursing towards “precision and risk pre-positioning” led to developing a more centralized nursing management model that integrates nursing resources, unifies management standards, and establishes a multi-dimensional risk prevention and control system, effectively improving the safety and continuity of nursing during complex diagnosis and treatment processes.^8^–^10^ However, current studies on centralized nursing management for pediatric bronchoscopic interventional therapy primarily focus on optimizing operational standards. The correlation between “risk prevention and control” and “symptom improvement”, and the quantitative evaluation of core indicators such as children’s postoperative airway function recovery and complication rate, remains unclear.^11^,^12^

This study aimed to evaluate the effectiveness of a centralized nursing management system in improving symptoms in children undergoing bronchoscopic interventional therapy. The results may provide a practical basis and theoretical support for improving nursing quality in this population of pediatric patients.

## METHODOLOGY

**Fig.1:**
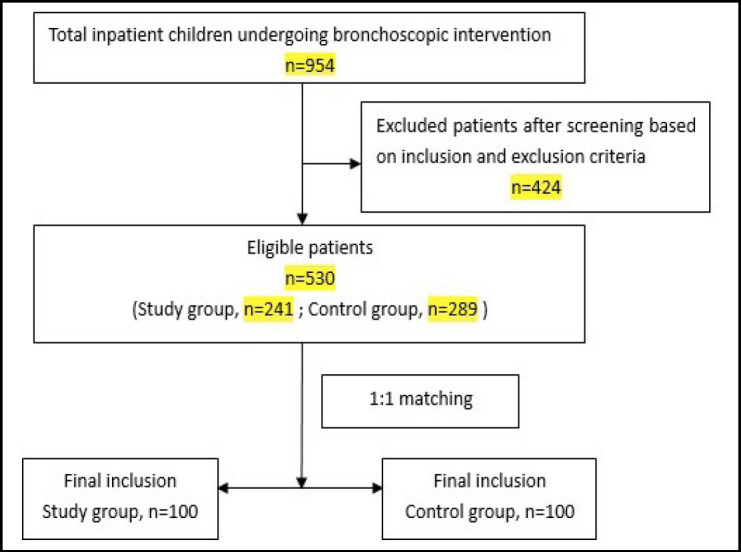
Flow chart of patient selection.

In this single-center retrospective comparative study, the clinical data of children undergoing bronchoscopic intervention admitted to Children’s Hospital of Hebei Province from January 2023 to December 2024 were retrospectively selected. The patients underwent bronchoscopic intervention and sedation according to the same institutional protocols, and other routine clinical treatments were kept consistent between the two groups as far as possible.. Patients were divided into a control group and a study group based on the nursing method (Fig.1).

### Inclusion Criteria:

- Aged 1–14 years.
- Clinically diagnosed as requiring bronchoscopic interventional therapy (e.g., airway foreign body removal, airway stenosis dilation, mucus plug clearance).
- Stable preoperative vital signs; ability to tolerate bronchoscopic interventional operation after evaluation.
- Complete clinical data, including preoperative examinations (blood routine, coagulation function, chest imaging), intraoperative operation records, and postoperative follow-up data, which could meet the statistical needs of research indicators.

### Exclusion Criteria:

- Severe dysfunction of the heart, liver, kidney, and other organs (e.g., decompensated congenital heart disease, acute liver injury, renal failure).
- Coagulation dysfunction or active bleeding.
- Allergic to drugs related to bronchoscopic interventional therapy (e.g., local anesthetics, hemostatic drugs).
- Complicated with severe infectious diseases or immune deficiency.
- Unable to cooperate with postoperative symptom assessment due to intellectual disability or communication disorders.

### Routine nursing:

*Preoperative nursing:* One day before the operation, the guardians of the children were verbally informed of the operation process, fasting and water prohibition time (four hours before the operation), and precautions. Preoperative examinations such as blood routine, coagulation function and chest imaging were completed, and first-aid drugs and instruments were prepared. Children and their families who showed signs of mental stress were given simple emotional comfort. At 30 minutes before the operation, atropine was intramuscularly injected as prescribed to reduce bronchial secretions, and 2% lidocaine was used for surface anesthesia of the nasal cavity and pharynx.

*Intraoperative nursing:* The children were assisted to take the supine position, and the bronchoscope was inserted through the nose. During the operation, the mental state, complexion, and the changes of heart rate and oxygen saturation displayed by the ECG monitor were closely observed. Oral secretions were removed in time to keep the airway unobstructed. If severe cough, bleeding, and other conditions occurred, symptomatic treatment was carried out, such as injecting 1:10,000 epinephrine to stop bleeding.

*Postoperative nursing:* After removing the scope, the children were instructed to stay in bed for 30 minutes and fast for three hours to prevent aspiration. The families were informed of the typical reactions that might occur after the operation, such as throat discomfort and blood in sputum. The respiratory rate, depth, and lip color were monitored. Oxygen inhalation (2–3 L/minutes) was given to those with dyspnea. Complications, such as hemoptysis and fever, were observed, and the doctor was notified promptly if any abnormality occurred. At discharge, a health manual was distributed, and the key points of family nursing were discussed.

*A centralized nursing management scheme based on risk prevention and control*: A professionally trained nursing team led by the head nurse and composed of responsible nurses, anesthetic nurses, and rehabilitation nurses was formed. A closed-loop management mode, consisting of “risk assessment - hierarchical intervention - whole-process monitoring - continuous nursing,” was established. The specific measures were as follows:

### Preoperative centralized risk prevention and preparation:

*Multidimensional risk assessment:* The Pediatric Airway Risk Assessment Scale (Peds-ARA) was used to assess airway-related risks such as airway stenosis and asthma history. The bleeding risk was determined based on blood routine (platelet count) and coagulation function (prothrombin time and activated partial thromboplastin time). The cooperation degree and emotional state of the children were assessed by the simplified version of the Revised Children’s Anxiety and Depression Scale (RCADS). Finally, combined with the results of the three items, the risk was divided into low, medium, and high, and personalized intervention schemes were matched.

*Centralized health education and psychological intervention:* Training was centrally carried out for children and their families in the education room. The operation process was explained through animation videos and doll demonstrations, and the key points of cooperation (such as avoiding head raising, head shaking, and mouth breathing) were emphasized. For children at high risk, picture books and family companionship were used to relieve their fear. One day before the operation, reminders about fasting and water prohibition, as well as preoperative preparation lists, were sent through the nursing APP to ensure the accurate transmission of information.

*Precise preoperative preparation:* First-aid items were prepared according to the risk level. Children at high risk were given preventive oxygen inhalation (3–4 L/min for 10 minutes) 30 minutes before the operation. For individuals with a history of asthma, bronchodilators (such as salbutamol, administered according to the doctor’s advice) were inhaled via atomization in advance of the operation, in accordance with the latest guidelines, to reduce the risk of airway spasm.

### Intraoperative precise cooperation and risk control:

All children received anesthesia or sedation according to the same institutional protocol. In the study group, enhanced intraoperative nursing cooperation and risk-based monitoring were implemented during the procedure.

### Hierarchical intraoperative monitoring:

Routine monitoring of heart rate and oxygen saturation was performed for children at low risk; Real-time monitoring of respiratory rhythm was added for children at medium and high risk, and early warning was given immediately when the oxygen saturation was < 90% or the heart rate fluctuation was > 20% of the baseline value.

### Refined operation cooperation:

When the bronchoscope was inserted into the supraglottic area and the central bronchial cavity, 2% lidocaine was injected in stages and stayed for adaptation to reduce the stimulation reaction. For children at high risk of bleeding or those requiring biopsy or other invasive bronchoscopic procedures, hemostatic medications and rescue measures were prepared in advance and administered when clinically indicated according to the physician’s order.. During the operation, the position of the instruments on the TV screen was closely observed to avoid lung injury caused by a severe cough.

### Postoperative centralized monitoring, complication prevention and control:

### Hierarchical rehabilitation monitoring:

The children were centrally placed in the postoperative observation area. The monitoring time for children at low risk was six hours and extended to 12 hours for children at medium and high risk. The Face, Legs, Activity, Cry, Consolability (FLACC) Scale was used to dynamically assess the degree of pain in young children who could not express themselves independently, or the Numerical Rating Scale (NRS) was used for children aged six years and older. Those with FLACC ≤ 3 or NRS ≤ 3 were given lozenges to relieve pharyngeal discomfort, while those with scores exceeding the standard received ibuprofen suspension and other medications as prescribed by the doctor. Respiratory function indexes were monitored every hour. The myocardial enzyme spectrum was rechecked once within six hours after the operation and again 12 hours after the operation for children at medium and high risk to identify abnormalities in cardiopulmonary load in a timely manner.

### Targeted treatment of complications:

When a small amount of hemoptysis (< 10 ml/24 h) occurred, the children were instructed to avoid forceful coughing. When the amount of hemoptysis was > 50 ml/24 hour, the head-down and feet-up position was immediately adopted, hemostatic drugs were rapidly infused, and vital signs were monitored to prevent shock. When bronchospasm occurred, oxygen inhalation was given immediately, and bronchodilators (salbutamol atomization inhalation) were administered, and the changes of wheezing in the lungs were auscultated until relieved.

### Continuous nursing and risk follow-up:

### Discharge risk assessment:

Before discharge, the improvement of symptoms and vital signs of the children was re-evaluated. Children were discharged only if they met the standards of “FLACC/NRS < 3, no active bleeding, and oxygen saturation ≥ 95%”.

### Postoperative continuous management:

Follow-up was conducted through the nursing APP within three days after discharge. The body temperature, expectoration, and diet of the children were recorded daily, and airway nursing skills were taught. Outpatient reexamination was conducted for children at medium and high risk seven days after the operation. Cardiopulmonary function recovery was assessed through chest auscultation and oxygen saturation detection, and any delayed complications were treated promptly.

### Observation Indicators:

### The following indices were collected from all patients:

### Treatment compliance of the two groups:

Pediatric adherence was categorized based on parent reports and nurse observations. Complete compliance: Children actively cooperated with diagnosis, treatment, and nursing, or followed health guidance after slight guidance, without rule violations affecting treatment. Partial compliance: Children had minor resistance or slight rule violations, which could be improved after intervention and had little impact on treatment and recovery. Non-compliance: Children continuously resisted diagnosis, treatment, and nursing, or had repeated rule violations with ineffective intervention, which might lead to delayed treatment and increased risk of complications.

Treatment compliance = (Number of complete compliance cases + Number of partial compliance cases) / Total number of cases × 100%.

### Vital signs of the two groups:

The vital signs, including oxygen saturation (SpO2), mean arterial pressure (MAP), and heart rate (HR), were recorded before and after intervention. Higher SpO_2_ is associated with a lower risk of hypoxemic episodes. Within the normal range, an appropriate decrease in MAP and HR typically indicates greater hemodynamic stability.

- Number of bronchoscopies, length of hospital stay, and hospitalization costs.
- Complication rate. The complications were recorded within 24 hours after operation.
- Nursing satisfaction of family members, assessed using the Likert 5-point scale (one point = Very dissatisfied, five points = Very satisfied). The evaluation content was designed from 4 core dimensions: communication experience, nursing professionalism, risk control, and humanistic care, with a total score of 100 points. The criteria were: Very satisfied (Total score ≥ 90), Satisfied (75–89), Average (60–74), Dissatisfied (< 60). “Very satisfied” and “Satisfied” were included in the total satisfaction. At pre-discharge stage, nursing staff outside the nursing team in this study distributed and collected the survey in anonymous to ensure the collection was independent of the nursing team in this study.

### Statistical analysis:

Data was analyzed by SPSS 25.0. Measurement data were described as (*x̄*+*s*) and tested by t-test; count data were expressed as frequency and composition ratio (%) and tested by χ^2^ test. Fisher’s exact test was used when the expected cell count was small. A P value < 0.05 was considered statistically significant.

## RESULTS

In this study, a total of 954 patients were initially enrolled, among whom 424 were excluded. Finally, 530 patients met the eligibility criteria for the present study, including 241 in the study group and 289 in the control group. The two groups were matched at a 1:1 ratio based on gender, age, type of disease, comorbidities, and ASA classification, resulting in 100 patients in each group (Fig.1). No statistically significant differences were observed in the demographic characteristics between the two groups (P>0.05) (Table-I).The treatment compliance of the study group (96.00%) was higher than that of the control group (87.00%) (P < 0.05) Table-II.

**Table-I:** Comparison of general data between the two groups.

Item	Study Group(n=100)	Control Group(n=100)	t/χ^2^Value	P Value
***Gender[n(%)]***				
Male	56(56.00)	52(52.00)	0.322	0.570
Female	44(44.00)	48(48.00)		
Age(*x̄* + *s*, years)	7.87±3.28	8.09±2.95	0.499	0.619
***Disease Type[n(%)]***				
Airway foreign body	38(38.00)	42(42.00)	0.379	0.945
Congenital airway stenosis	25(25.00)	23(23.00)		
Severe pneumonia	22(22.00)	20(20.00)		
Others	15(15.00)	15(15.00)		
***Comorbidities[n(%)]***				
None	68(68.00)	72(72.00)	0.440	0.932
Mild anemia	18(18.00)	15(15.00)		
Malnutrition	10(10.00)	9(9.00)		
Others	4(4.00)	4(4.00)		
***ASA Classification[n(%)]***				
Grade I	52(52.00)	55(55.00)	0.330	0.848
Grade II	45(45.00)	43(43.00)		
Grade III	3(3.00)	2(2.00)		

**Table-II:** Comparison of treatment compliance between the two groups [n (%)].

Group	Number of Cases	Complete Compliance	Partial Compliance	Non-Compliance	Treatment Compliance
Study Group	100	59(59.00)	37(37.00)	4(4.00)	96(96.00)
Control Group	100	46(46.00)	41(41.00)	13(13.00)	87(87.00)
*χ^2^Value*					5.207
*P Value*					0.022

Before the intervention, there were no significant differences in SpO_2_, MAP, and HR between the two groups (P > 0.05). After the intervention, SpO_2_ levels in both groups increased compared to those before the intervention, while MAP and HR decreased; the study group’s indicators were better than those of the control group (P < 0.05; Table-III). As demonstrated in Table-IV, the number of bronchoscopic procedures and hospitalization costs were lower, and the length of hospital stay was shorter in the study group compared to the control group (P < 0.05). The incidence of complications was numerically lower in the study group than in the control group (2.00% vs. 9.00%), although the difference did not reach statistical significance when Fisher’s exact test was applied (P = 0.058; Table-V). Family satisfaction was numerically higher in the study group than in the control group (97.00% vs. 90.00%), but this difference did not reach statistical significance when Fisher’s exact test was applied (P = 0.082; Table-VI).

**Table-III:** Comparison of Vital Signs between the two groups (*x̄*+*s*).

Time	Group	Number of Cases	SpO_2_ (%)	MAP (mmHg)	HR (times/min)
Before Intervention	Study Group	100	94.07±1.62	86.94±4.17	118.95±8.32
	Control Group	100	93.71±2.04	87.20±4.45	120.05±9.13
	*t* Value		1.382	0.426	0.891
	*P* Value		0.169	0.670	0.374
After Intervention	Study Group	100	98.62±1.36	78.21±3.35	95.24±5.26
	Control Group	100	96.91±1.55	82.42±3.76	99.71±6.41
	*t* Value		8.293	8.360	5.391
	*P* Value		0.000	0.000	0.000

**Table-IV:** Comparison of the number of bronchoscopic procedures, length of hospital stay, and hospitalization cost between the two groups (*x̄* + *s*).

Group	Number of Cases	Number of Bronchoscopies (times)	Hospitalization Cost (Yuan)	Length of Hospital Stay(d)
Study Group	100	1.21±0.39	7541.21±1137.32	8.41±2.06
Control Group	100	1.59±0.54	8671.43±1245.53	9.79±3.18
*t Value*		5.705	6.701	3.642
*P Value*		0.000	0.000	0.000

**Table-V:** Comparison of complication rates between the two groups [n(%)].

Group	Number of Cases	Mucosal Bleeding	Laryngeal Edema	Irritative Cough	Pneumothorax	Total Incidence
Study Group	100	0(0.00)	1(1.00)	0(0.00)	1(1.00)	2(2.00)
Control Group	100	2(2.00)	3(3.00)	3(3.00)	1(1.00)	9(9.00)
*P* Value						0.058^a^

***Note:***

a P value was calculated using Fisher’s exact test.

**Table-VI:** Comparison of Nursing Satisfaction of Children’s Family Members between the two groups [n(%)].

Group	Number of Cases	Very Satisfied	Satisfied	Average	Dissatisfied	Total Satisfaction
Study Group	100	59(59.00)	38(38.00)	2(2.00)	1(1.00)	97(97.00)
Control Group	100	51(51.00)	39(39.00)	7(7.00)	3(3.00)	90(90.00)
*P* Value						0.082^a^

***Note:***

a P value was calculated using Fisher’s exact test.

## DISCUSSION

The role of centralized nursing management plan based on risk prevention and control in improving treatment compliance. Children’s fear of medical procedures is the core reason for low treatment compliance, and simple verbal comfort in routine care is difficult to effectively alleviate children’s anxiety. In this study, the treatment compliance of the research group (96.00%) was significantly higher than that of the control group (87.00%) (P<0.05). The reason for this is that the centralized nursing management plan focused on both layered intervention and humanistic care. Preoperative assessment of emotional status was combined with the Children’s Anxiety Scale, and cognitive characteristics of young children were adapted through animated propaganda and picture book guidance. At the same time, family members were encouraged to participate in preoperative preparation, psychological reassurance, and perioperative communication, which helped reduce fear of unfamiliar environments; Adjust the communication frequency according to the risk level during the operation, such as providing verbal comfort to high-risk children every five minutes, and enhancing their sense of control through pre operation warnings (such as “there will be some itching next, just hold on for a while”); Timely intervention of discomfort through pain assessment after surgery can reduce subsequent nursing resistance caused by pain. This is consistent with the research findings of Xiaona L et al.,^13^ which used specialized nursing interventions for fiberoptic bronchoscopy in children. The children’s cooperation was significantly improved, and their heart rate and respiratory rate remained stable during the examination period, confirming the need for a combination of behavioral guidance and emotional comfort in pediatric medical care; This study is highly consistent with the research of Smith W et al.,^14^ who proposed that a nursing model with deep involvement of family members is associated with better compliance among pediatric patients. Furthermore, this study further refined the psychological intervention methods for young pediatric patients, making up for the lack of targeted humanistic interventions in some studies.

The regulatory value of centralized nursing management plan based on risk prevention and control on vital signs and complications. In bronchoscopy intervention therapy, airway stimulation and anesthesia stress can easily lead to a decrease in SpO_2_, an increase in HR and MAP. Conventional nursing often intervenes after abnormal indicators, which can delay the optimal treatment timing. This study showed that after intervention, the SpO_2_ was significantly higher in the study group than in the control group, while HR and MAP were closer to the normal range. The incidence of complications was numerically lower in the study group than in the control group (2.00% vs. 9.00%), but this difference did not reach statistical significance after Fisher’s exact test. The core mechanism lies in the risk prevention and control design of centralized nursing: preoperative risk levels were divided by the children’s airway risk assessment scale and coagulation function indicators, and high-risk children were given prophylactic oxygen therapy and hemostatic drugs in advance; During the operation, increase airway pressure monitoring for children with medium to high risk. When SpO_2_ <90% or HR fluctuation>20% of the baseline value, immediately warn and cooperate with staged anesthesia to reduce airway stimulation; Postoperative centralized monitoring for 6-12 hours, targeted treatment for complications such as hemoptysis (>50ml/24h) and airway spasm. Wu Xudan’s study adopted a risk prevention and control hierarchical nursing approach, effectively reducing discomfort, while this study further reduced risk through a closed-loop prevention and control model; Compared with the study by Swift JA et al.,^15^ this study focuses more on real-time risk warning during surgery, while foreign studies tend to focus more on postoperative follow-up interventions. This difference is due to the higher clinical demand for rapid emergency response during surgery in pediatric medical institutions in China, and this research plan is more in line with domestic clinical practice.

Optimization effect of centralized nursing management plan based on risk prevention and control on diagnosis and treatment efficiency and health economics. The efficient utilization of medical resources is an important consideration in pediatric nursing. In this study, the research group had fewer bronchoscopes, shorter hospital stays, and lower hospitalization costs (P<0.05), reflecting the efficient and collaborative advantages of centralized nursing management. Preoperative risk assessment (such as airway stenosis degree) was conducted to avoid repeated endoscopic examinations due to patient intolerance and operational risks; After surgery, through graded rehabilitation monitoring (low-risk six hours, medium high risk 12 hours), the nursing intensity is dynamically adjusted. For example, low-risk children who recover quickly can be guided to eat independently in advance to accelerate the rehabilitation process; At the same time, although centralized nursing requires the investment of specialized nursing teams, improved process coordination, fewer repeated procedures, and more efficient resource utilization may contribute to lower total hospitalization expenses; Compared with the study by Charles M Bergman et al.,^16^ this study focuses more on the efficient integration of medical resources, while foreign research focuses on the impact of home care training on postoperative rehabilitation. This study fills the research gap in the health economics dimension of pediatric bronchoscopy nursing and provides a scalable nursing path for cost control of pediatric minimally invasive diagnosis and treatment. Nevertheless, differences in length of hospital stay and hospitalization cost are multifactorial, and future prospective or multicenter studies should be conducted for further confirmation.

Improvement mechanism of centralized nursing management plan based on risk prevention and control on family satisfaction. Family satisfaction is an important feedback indicator for the quality of pediatric nursing. The family satisfaction rate was numerically higher in the study group than in the control group (97.00% vs. 90.00%), although this difference did not reach statistical significance after Fisher’s exact test. The key is that the centralized nursing management plan meets the core needs of family members: firstly, the right to be informed of risks and cooperation points before surgery, real-time feedback on the patient’s situation during surgery, and daily push of recovery progress after surgery, to avoid anxiety caused by information asymmetry; The second is the right to participate in decision-making, such as communicating with family members in advance and respecting their opinions on key decisions related to child care; The third is professional trust, which enhances the recognition of nursing quality by family members through proficient operation and timely abnormal handling, which is consistent with the consensus proposed by SmithBattle L et al.^17^ that “family centered nursing can improve family satisfaction”; The advantage of this study lies in further clarifying the satisfaction path of the three core needs of family members, namely “information awareness, decision-making participation, and professional trust”, which makes up for the lack of detailed analysis of family needs in some studies. It suggests that pediatric nursing needs to attach importance to the collaboration between family members and medical staff, and build trust relationships through effective communication and professional services.

The supplementary value of this study to medical literature mainly lies in two aspects: firstly, it enriches the research dimensions of pediatric bronchoscopy nursing. Previous studies at home and abroad have mainly focused on clinical efficacy and safety indicators. This study adds dimensions of health economics and family needs subdivision, improving the nursing research system; The second is to provide a practical path that is suitable for pediatric clinical practice in China, without relying on high-end equipment, and can be implemented by integrating existing resources, solving the adaptability problem of foreign nursing models in primary hospitals in China.

### Strengths:

The advantage of this study lies in its strong targeted approach, which is in line with the cognitive and airway characteristics of young children; Comprehensive intervention dimensions, taking into account medical risks, psychological needs, and economic costs; High practical feasibility, easy to promote in pediatric medical institutions at all levels.

### Limitations:

It includes its retrospective, single-center design, which may introduce confounding and selection bias. Second, group comparability may have been limited because only five baseline variables were compared between the groups, and residual confounding from unmeasured clinical factors cannot be excluded. Thirdly, a ceiling effect may exist in the satisfaction assessment as the nursing satisfaction of family members was assessed using the Likert 5-point scale, and pediatric adherence was categorized based on parent reports and nurse observations, which may have subjective bias. In addition, the numbers of complication events and non-satisfied cases were small, which limited the statistical power for detecting between-group differences in these outcomes. Fourthly, the follow-up period is relatively short, and the long-term airway function recovery of the patient after surgery has not been tracked. Fifthly, the intervention differences among children of different ages and disease types have not been refined. In the future, the sample size can be expanded to conduct multicenter studies, extend the follow-up period to evaluate long-term effects, and refine personalized nursing plans to further improve intervention accuracy.

## CONCLUSION

Centralized nursing management based on risk prevention and control was associated with improved treatment compliance, more stable perioperative vital signs, fewer bronchoscopic procedures, shorter hospital stay, and lower hospitalization costs in children undergoing bronchoscopic interventional therapy. The observed numerical reduction in complications and improvement in family satisfaction should be interpreted cautiously and require further confirmation in larger prospective studies.

### Author’s contributions:

**YC and WW:** Literature search, study design and manuscript writing. **PH, LH and YW:** were involved in data collection, data analysis and interpretation. Critical Review. **YC and WW:** Manuscript revision and validation and is responsible for the integrity of the study. All authors have read and approved the final manuscript.
